# Patients’ Satisfaction with the quality of online versus in-person DBT skills group therapy: A pilot study

**DOI:** 10.1192/j.eurpsy.2022.1824

**Published:** 2022-09-01

**Authors:** A. Abdelkarim, I. Abdelfattah

**Affiliations:** Alexandria Faculty of Medicine, Psychiatry, Alexandria, Egypt

**Keywords:** DBT, borderline personality disorder, Online Therapy

## Abstract

**Introduction:**

Since the beginning of the COVID-19 era, there has been a major shift of psychiatry and psychotherapy practice to the online venues, or what has been broadly known as telepsychiatry. A practice that has been very practical since then. And yet, there has been a debate about the patients’ degree of satisfaction with the therapeutic process, especially with a modality like group therapy, which has not been widely researched.

**Objectives:**

The objective of this pilot is to assess the level of patients’ satisfaction among both online and in-person participants of dialectical behavioral therapy (DBT) skills group as a part of comprehensive outpatient DBT program.

**Methods:**

27 DBT skills group participants completed an online form including demographic data, type and duration of group attended, in addition to the Arabic version of the Satisfaction with Therapy and Therapist Scale- Revised (STTS-R).

**Results:**

The majority of the 27 participants were females (88.9%), of which 81.4% were 18-34 years old and 77.4 % at least had a university degree. Among all the participants, 63% were online group attendants versus 37% in-person. The mean total of patient’s satisfaction with the in-person group was 53.5 in comparison to 49.2 in online group participants. Also, 90% of in-person group participants reported that the group helped them in dealing with presenting problem to an extent in comparison to 82.2% of online participants.

**Conclusions:**

Although the COVID-19 pandemic mandated more use of telepsychiatry, in-person DBT skills group participants reported higher satisfaction of their therapy in comparison to online group participants.

**Disclosure:**

No significant relationships.

